# Research on the Influence of Liquid Metal Embrittlement Cracks on the Strength and Fatigue Life of Spot-Welded Joints of Galvanized Q&P980 Steel

**DOI:** 10.3390/ma17246149

**Published:** 2024-12-16

**Authors:** Jie Zou, Huayang Xiang, Zhenfei Zhan, Wenbo Zhuo, Li Huang, Yuxiang Ji, Qing Liu

**Affiliations:** 1 School of Mechanical and Electrical and Vehicle Engineering, Chongqing Jiaotong University, Chongqing 400074, China; zoujie@mat-jitri.cn (J.Z.); 980251006xyf@gmail.com (H.X.); hli@jitri-amrd.com (L.H.); 2 Materials Bigdata and Applications Division, Materials Academy Jitri, Suzhou 215131, China; zhuowb@mat-jitri.cn (W.Z.); jiyuxiang@mat-jitri.cn (Y.J.); liuqing@mat-jitri.cn (Q.L.); 3 School of Mechanical Engineering, Nanjing University of Science and Technology, Nanjing 210094, China; 4 Key Laboratory for Light-Weight Materials, Nanjing Tech University, Nanjing 210009, China; 5 School of Advanced Technology, Xi’an Jiaotong-Liverpool University, Suzhou 215123, China

**Keywords:** liquid metal brittleness, resistance spot welding, fatigue test, finite element analysis

## Abstract

Galvanized high-strength steel has emerged as a key focus in automotive lightweighting research. During resistance spot welding of galvanized steel, the phenomenon of liquid metal embrittlement (LME) can occur, which is characterized by the appearance of irregular cracks on the weld spot surface. However, the impact of LME cracks on the mechanical properties of joints remains unclear. This study investigates the LME phenomenon and its effects on the performance of spot-welded joints using galvanized QP980 steel as the subject. By combining theoretical analysis, experimental methods, and simulations, the formation and characteristics of LME cracks are explored through resistance spot welding experiments and elemental analysis. The influence of LME cracks on the static strength of joints is assessed through quasi-static tensile tests, fracture surface analysis, and theoretical calculations. Finite element simulations of the static tensile process reveal that LME cracks alter the stress–strain fields during joint failure. Additionally, the study examines how the location and size of LME cracks influence these effects. Finally, fatigue testing and fracture analysis of spot-welded joints demonstrate that LME cracks can negatively impact the fatigue life of joints.

## 1. Introduction

Automotive lightweighting has become a key development trend for improving energy efficiency and reducing emissions. This approach involves reducing the curb weight of vehicles while maintaining strength and safety performance, thereby enhancing vehicle dynamics and achieving energy savings and emission reductions [1]. Compared to traditional carbon steel, the use of advanced high-strength steel (AHSS) with both high strength and high ductility is one of the most effective strategies for achieving automotive lightweighting. Quenching and partitioning steel, a representative of third-generation AHSS, has been widely adopted in vehicle body manufacturing [2]. To prevent oxidation and corrosion of the steel surface, galvanizing is commonly applied as a protective treatment [3].

In automotive body structure assembly, resistance spot welding (RSW) is the primary joining technology due to its high efficiency and reliable weld quality [4]. During the RSW process of galvanized high-strength steel, the zinc coating, which has a much lower melting point than the steel substrate, melts first. The molten zinc infiltrates the steel substrate and, under the stress applied by the electrodes, leads to the formation of liquid metal embrittlement (LME) cracks [5], which are irregular cracks on the surface of the weld. These LME cracks compromise the integrity of the joint, adversely affecting its toughness and, consequently, its service performance. Górka J et al. [6] investigated the impact of varying laser welding parameters on the structure, mechanical properties, and corrosion resistance of welded joints in 1.2 mm low-carbon steel galvanized sheets. They found that changes in welding energy significantly affected weld width, hardness, and tensile properties, while all joints exhibited good corrosion resistance and ductility.

To investigate the impact of LME cracking on spot-welded joint performance, researchers typically employ comparative experiments using galvanized and non-galvanized steel sheets. The tensile–shear test is a commonly used method for evaluating the static strength of welded joints due to its simple structure and widespread application in welded structural designs subjected to tensile–shear loads [7]. Digiovanni et al. [8] studied the effect of LME on joint strength in three different types of high-strength steels. They found that TRIP1100 steel joints experienced the most significant decrease in strength, with numerous cracks observed on the weld surface and LME cracks present in the fracture path. Ge Xiaomin [9] obtained similar results when investigating the impact of LME on the static strength of QP1180 steel joints. As the loading displacement increased, LME cracks extended simultaneously with failure propagation cracks, ultimately leading to weld failure when they intersected. Based on these findings, some researchers have proposed that when the weld crack length exceeds a certain critical value, it may affect joint strength. Choi et al. [10] experimentally observed that TRIP steel joint strength did not significantly decrease when LME cracks were smaller than 325 μm; however, strength noticeably declined when cracks exceeded 500 μm. Digiovanni et al. [11] confirmed this hypothesis through finite element simulations, discovering that crack lengths exceeding 350 μm significantly impacted strength loss. Related studies indicate that longer crack lengths have a greater influence on joint static strength. This effect is particularly pronounced in high-strength steels with higher tensile strengths, where LME cracks have a more significant impact on joint static strength. However, the underlying reasons for this phenomenon remain unclear and require further investigation and supplementation by future researchers.

Considering fatigue and finite element analysis, the impact of LME cracks on joint fatigue life is primarily assessed by observing the correlation between fatigue crack propagation paths and LME cracks. Current research on the effect of LME cracks on joint fatigue life mainly focuses on how different types of LME cracks influence fatigue life. Kim et al. [12] found that an LME crack is located directly above the indentation, making it a Type A crack which has little effect on the fatigue life of the joint. Type B cracks are cracks located on the surface of the weld at the shoulder of the indentation. Fatigue cracks typically initiate at the sheet interface tip and propagate along the sheet thickness direction, generally not passing through the Type A crack region. Jang et al. [13] investigated the influence of LME cracks on fatigue life in TRIP1180 steel sheets using tensile–shear (TS) and cross-tension (CT) specimens. Even when crack depth exceeded 45% of the sheet thickness, fatigue life did not significantly decrease. Thus, the effect of Type B cracks on high-cycle fatigue behavior can be considered negligible. Simulation analysis revealed that under fatigue loading conditions, these cracks neither alter the location of maximum local stress nor induce stress concentration. Wang et al. [14] discovered through fatigue testing of spot-welded joints that Type B cracks do not affect the fracture process during fatigue tests. This is because fatigue cracks and LME cracks are located on opposite sides, and the fatigue crack propagation process is unrelated to LME cracks. Kwon et al. [15] studied high-cycle fatigue behavior of TRIP steel under TS and CT loading modes. In addition to observing Type C cracks, they also noted the presence of “pre-cracks” at the sheet interface. Under cyclic loading, their influence varied depending on the loading mode. In the TS loading mode, only “pre-cracks” participated in the joint fatigue fracture process, while in the CT loading mode, LME cracks in some specimens contributed to fatigue fracture propagation.

This study investigates the impact of LME cracks, formed during the resistance spot welding of galvanized QP980 steel, on the mechanical properties of joints. The characteristics of LME cracks were observed through spot welding process experiments. The effect of LME cracks on the static strength of the joints was preliminarily evaluated through quasi-static tensile tests and fracture surface analysis. The underlying mechanisms were further elucidated using material failure models and finite element simulations. The study also explored the effects of LME cracks located at different positions. Finally, the influence of LME cracks on the fatigue life of joints was examined.

## 2. Testing Process

### 2.1. Experimental Materials and Methods

The materials used in this study consisted of 1.2 mm thick QP980 steel, both in galvanized and uncoated forms. The galvanized steel had a coating thickness of 13 µm, applied via electroplating. Table 1 provides the chemical composition of QP980 steel, while Table 2 details its mechanical properties. The microstructure of QP980 steel is primarily composed of martensite, ferrite, and retained austenite.

**Figure 1:**
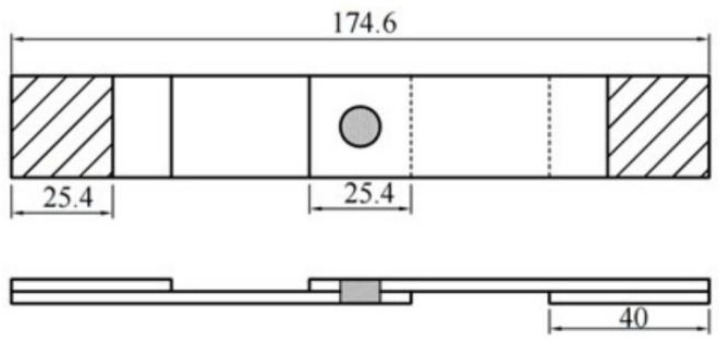
Schematic diagram of the size of the tensile shear specimen.

The resistance spot welding tests were conducted using a KUKA robotic system equipped with an OBARA medium-frequency inverter DC welding gun. Standard Cu-Cr electrodes with the specification F1-16-20-8-6.5 were used, and the electrodes had a cross-sectional diameter of 6 mm. The welding parameters are detailed in Table 3. The spot welding tests were divided into process and mechanical tests, with each type performed in triplicate. The mechanical tests included quasi-static tensile tests and fatigue tests, with the tensile–shear test specifications illustrated in the accompanying Figure 1. The quasi-static tensile tests were carried out using an Instron 5982 universal testing machine at a tensile rate of 10 mm/min, with a gauge length of 50 mm, as measured by an extensometer. Load–displacement data were recorded throughout the test. The fatigue tests were performed using an Instron 10,000 low-force dynamic–static material testing machine, with a loading frequency of 30 Hz and a stress ratio of 0.1.

### 2.2. LME Crack Observation

**Figure 2:**
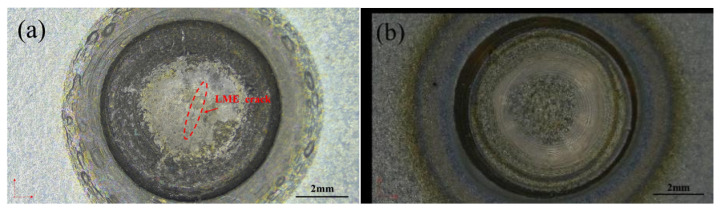
Q&P980 steel spot welding joint surface observation. (**a**) A galvanized layer. (**b**) No galvanized layer.

During the resistance spot welding process, LME (liquid metal embrittlement) cracks formed were examined through both surface and cross-sectional observations using a stereomicroscope. The results of the surface observation of the weld points are shown in Figure 2. It is suggested that the zinc coating in the central region of the weld point melted first, extruded under electrode pressure, and spread to the indentation step, thus covering the cracks in that area. As a result, only cracks directly above the molten pool were observed. LME cracks were only observed on the surfaces of the galvanized steel weld points; no cracks were found on the uncoated steel, indicating that the presence of the zinc coating is a significant factor contributing to the formation of LME cracks during the resistance spot welding process.

**Figure 3:**
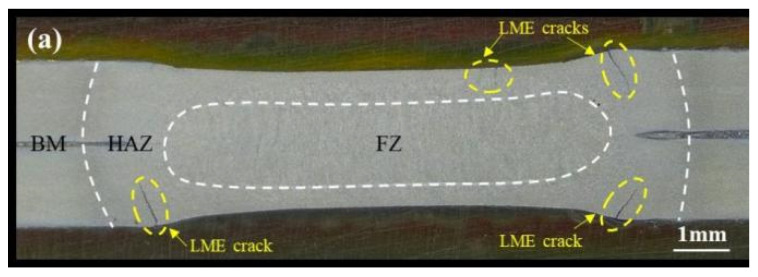

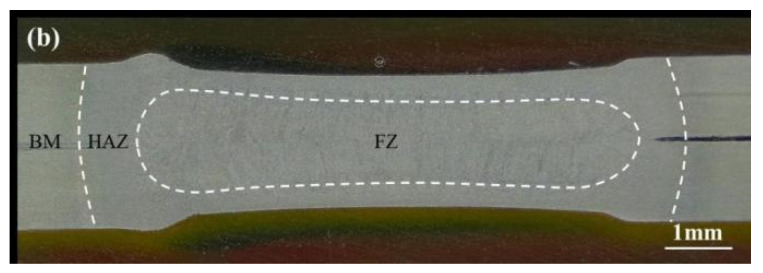
Q&P980 steel spot welding joint profile metallographic diagram. (**a**) A galvanized layer. (**b**) No galvanized layer.

The samples were sectioned along the axis of the weld point and prepared for microscopy through embedding, grinding, polishing, and etching. The prepared samples were then observed under a stereomicroscope. The cross-sectional observations of the spot welds are shown in Figure 3. Examination of the cross-sections revealed that cracks in the galvanized samples were primarily distributed above the molten pool area (Type A) and at the edges of the indentation steps (Type B); there were no Type C cracks. This region is subject to complex temperature and stress field variations, which facilitate crack formation. In contrast, no cracks were observed in the cross-sections of the uncoated steel samples, further highlighting the critical role of the zinc coating in the formation of LME cracks. Additionally, the average diameters of the molten pools for galvanized and uncoated samples were measured as 6.34 mm and 6.98 mm, respectively.

**Figure 4:**
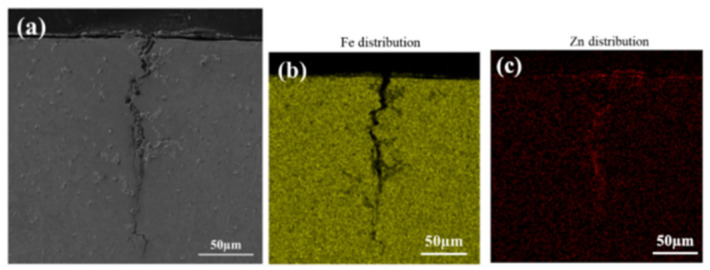
EDS analysis of cracks above the fusion zone located in the upper right corner of the section (Type A).(**a**) EDS scanning. (**b**) Fe distribution. (**c**) Zn distribution.

**Figure 5:**
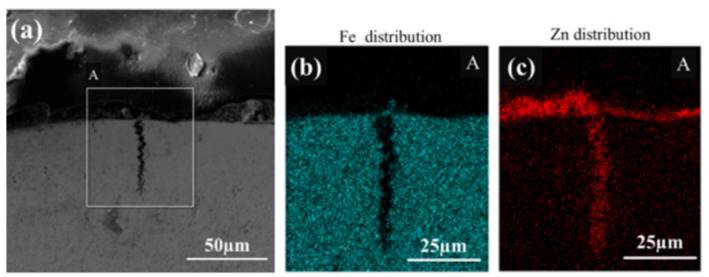
EDS analysis of cracks at the step of the heat-affected zone located directly above the section (Type B).(**a**) EDS scanning. (**b**) Fe distribution. (**c**) Zn distribution.

**Figure 6:**
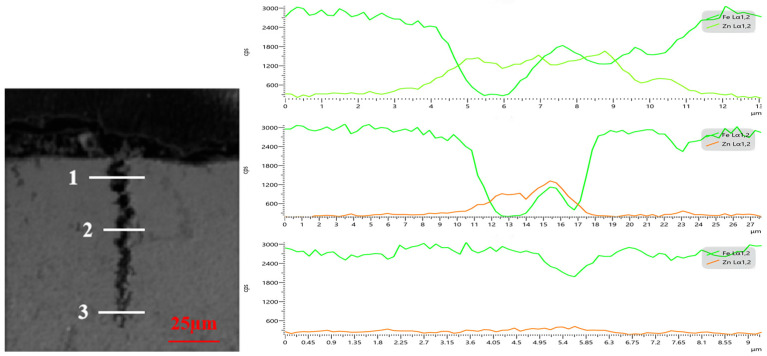
LME crack area line scan results.

EDS analysis of LME cracks and their surrounding elemental distribution was conducted using a scanning electron microscope. Figure 4 and Figure 5 show the EDS analysis results for cracks located above the molten pool (Type A) and at the steps of the heat-affected zone (Type B), respectively. The surface scan results reveal that a significant amount of Zn accumulates at the steps of the heat-affected zone during welding, which also corresponds to the area of electrode stress concentration. Consequently, the Zn concentration in the cracks at these steps is higher than that observed above the molten pool. Further line scan analysis of the cracks at the heat-affected zone steps, as shown in Figure 6, indicated that the Zn content in the cracks decreases with increasing crack depth.

### 2.3. Three Dimensional Scanning of Solder Joints in Mechanical Specimens

**Figure 7:**
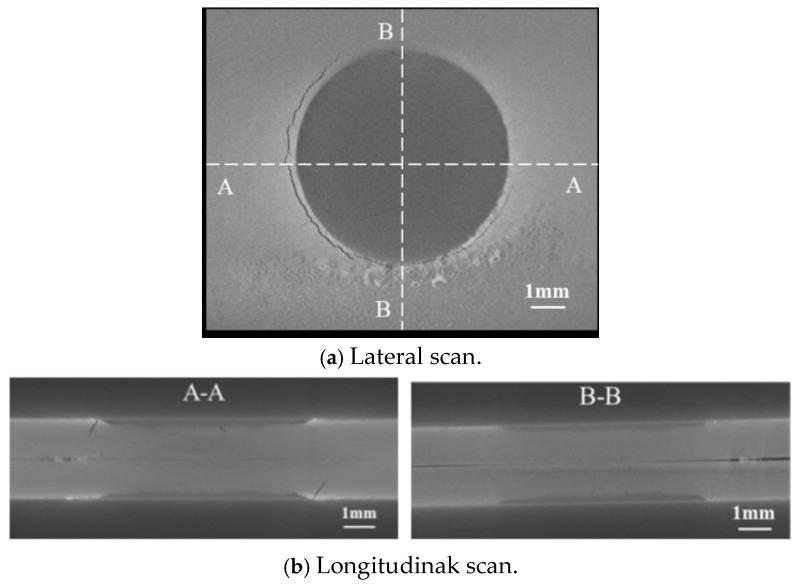
CT scans of LME cracks in spot-welded joints.

To comprehensively determine the location and depth of LME cracks in the galvanized samples, three-dimensional Computed Tomography (CT) scanning was performed on the weld points and surrounding areas of the tensile specimens prior to conducting quasi-static tensile tests. This also provided a foundation for analyzing joint failure processes and establishing simulation models. Figure 7 presents the scan results of the specimens. Both the upper and lower plates exhibit LME cracks, which are primarily located at the edges of the heat-affected zone steps. These cracks are arc-shaped and show varying depths.

**Figure 8:**
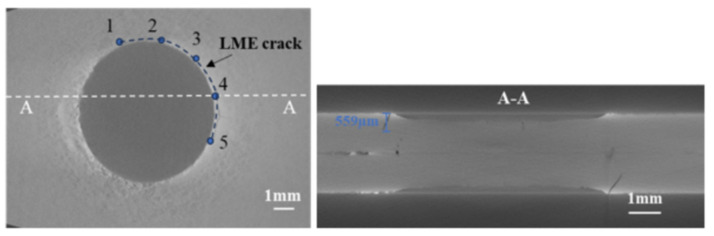
LME crack depth measurement.

**Figure 9:**
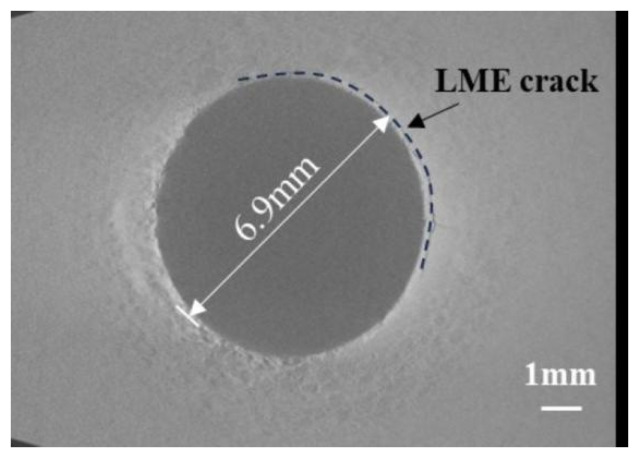
LME crack position diameter measurement diagram.

To further evaluate the sensitivity of LME cracks in the joints and to lay the groundwork for simulating these cracks, the average crack length is defined as an indicator of crack sensitivity. The average crack length is determined by measuring the depth of cracks at five points along the surface crack propagation path using CT scan results and then averaging these measurements. Figure 8 illustrates the measurement scheme. In addition to determining the specific depths of various LME crack types, it is also necessary to measure the crack diameters. Figure 9 shows the measurement of the approximate diameters of LME cracks. Table 4 summarizes the diameters of LME cracks at the upper and lower plates of galvanized TS and CP specimens, with crack diameters generally clustered around 7 mm.

## 3. Material Failure Model

Fracture strain is a critical criterion for assessing material failure and is typically obtained through material testing. Stress triaxiality, a commonly used stress state parameter, reflects volumetric and shape changes within a material’s internal structure. Areas with high stress triaxiality values generally exhibit less plastic deformation, often indicating regions with significant volumetric deformation that can release a considerable amount of elastic strain energy, leading to severe stress concentration. Conversely, areas with low stress triaxiality are more prone to shear fracture.

**Figure 10:**
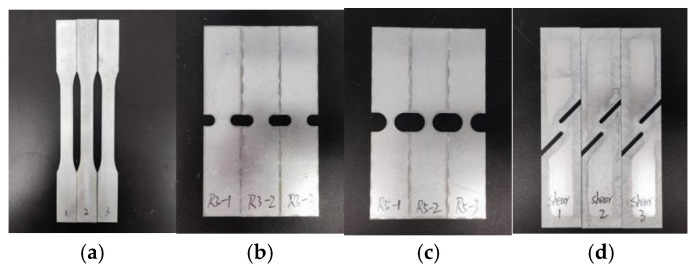
Material test specimens. (**a**) Uniaxial tensile test specimen. (**b**) R3 notch specimen. (**c**) R5 notch specimen. (**d**) Shear specimen.

**Figure 11:**
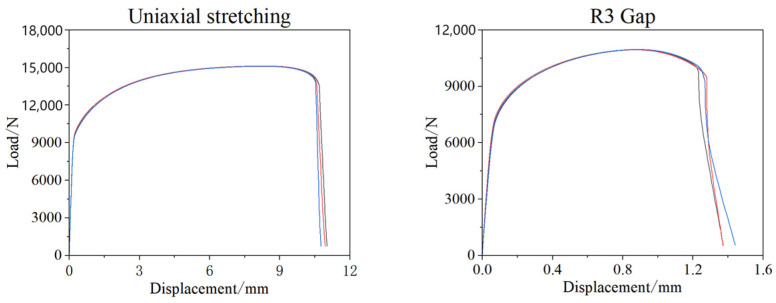

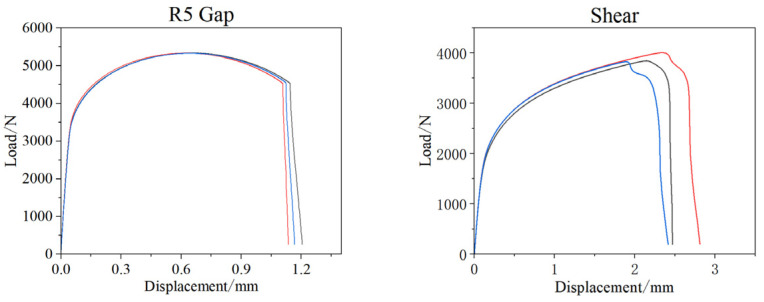
Test load–displacement results for each specimen.

To obtain the fracture strain of the material under different stress states, four types of quasi-static tensile tests were designed according to sample standards. The geometries of the samples are shown in Figure 10. The material selected for these tests was galvanized Q&P980 steel, and the load–displacement results are presented in Figure 11.

**Figure 12:**
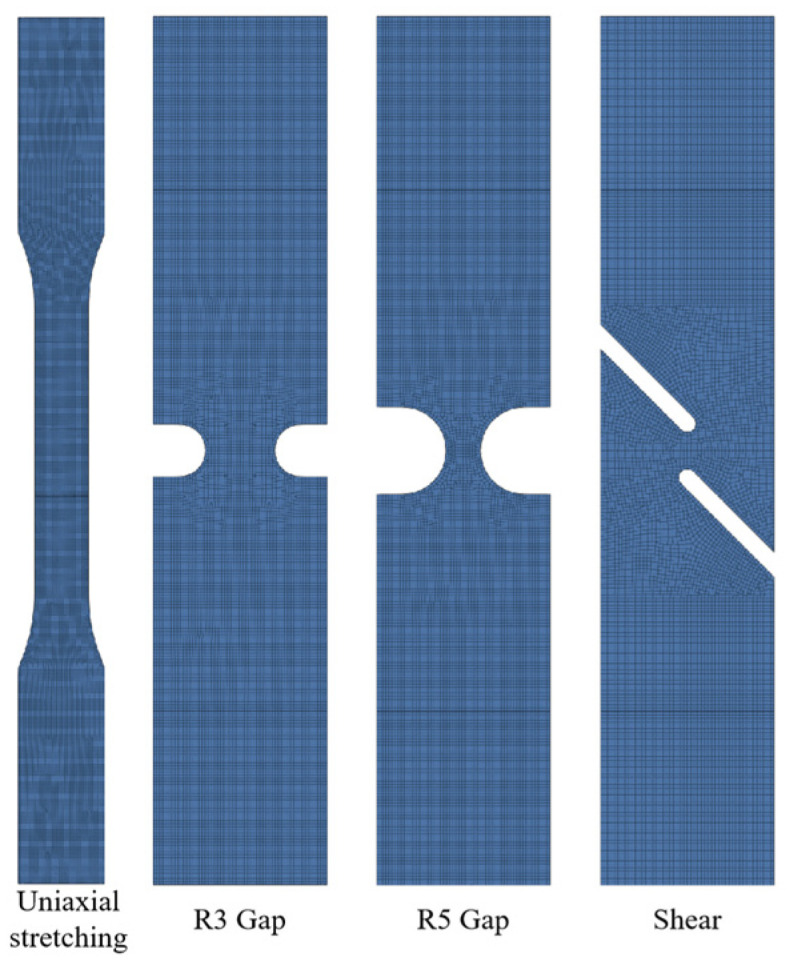
Finite element simulation model of each material test specimen.

The Gissmo model is widely used for predicting fracture failure behavior and has been incorporated into LS-DYNA. This model is a linear damage accumulation failure model that defines a damage variable. The initial damage is zero, and as loading continues, the material’s plastic strain increases, leading to gradual accumulation of damage. The damage variable increases until it reaches a value of 1, at which point the material fractures. In this study, the Gissmo failure model’s fracture limit criterion was used as the basis for failure assessment. Combining experimental data with finite element simulations, finite element models were first established for the aforementioned samples, as shown in Figure 12. The Swift–Ludwik hardening criterion, as described by Equation (1) [16], was employed. By continuously adjusting the hardening criterion, the finite element models were optimized to closely match the load–displacement results obtained from the experiments.

(1) $$\sigma =(1-\alpha )A{\left(\varepsilon_{p}+\varepsilon_{0}\right)}^{n}+\alpha (\sigma_{s}+C\varepsilon_{p}^{n})$$

where

*σ* represents the flow stress;
*α* is the weight coefficient;
*ε*_p_ denotes the plastic strain;
*ε*_0_ is the plastic strain corresponding to the yield strength, also known as the yield strain;
*σ*_s_ indicates the yield strength;
*A*, *C*, *m*, and n are the material parameters fitted in the model.

**Figure 13:**
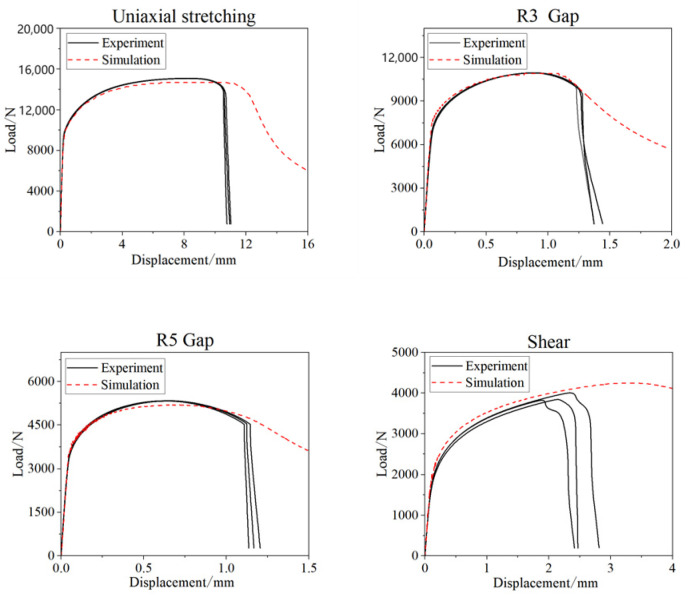
Comparison of test and simulation results for each specimen.

By continuously optimizing the weight coefficient, when α = 0.23, the load–displacement results from the experiments and simulations are shown in Figure 13. It can be observed that the fit between the two is quite good. Once the results were closely aligned, the key elements’ damage values could be extracted from the finite element simulations, along with their corresponding triaxial stress values and equivalent plastic strain values. By calculating these, the relationship between stress triaxiality and fracture strain for different tensile specimens could be determined, as presented in Table 5.

**Figure 14:**
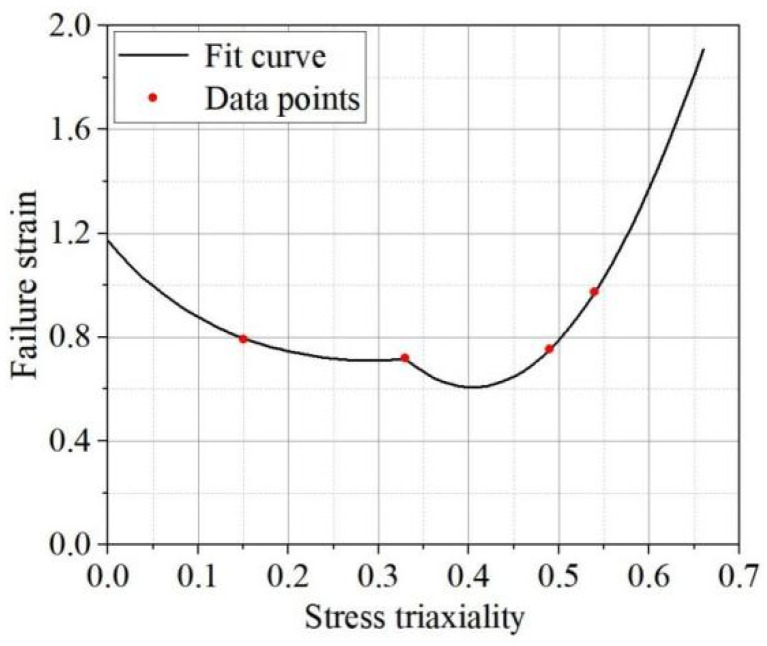
Fracture strain-versus-stress triaxiality curve-fitting result plot.

The obtained data on stress triaxiality and failure strain were fitted in segments, focusing on fracture behavior within the range of stress triaxiality from 0 to 0.66. For stress triaxiality in the range of 0 to 0.33, the Hosford–Coulomb fracture failure model was used for fitting. This model, proposed by Mohr [17], is an extended Mohr–Coulomb criterion developed for defect materials. For the range of 0.33 to 0.66, polynomial fitting was employed. Figure 14 shows the fitted curve of fracture strain versus stress triaxiality. This established the failure model for the base material, which served as the foundation for subsequent quasi-static tensile simulations.

## 4. Results and Discussion

### 4.1. Analysis of Static Tensile Test Results

**Figure 15:**
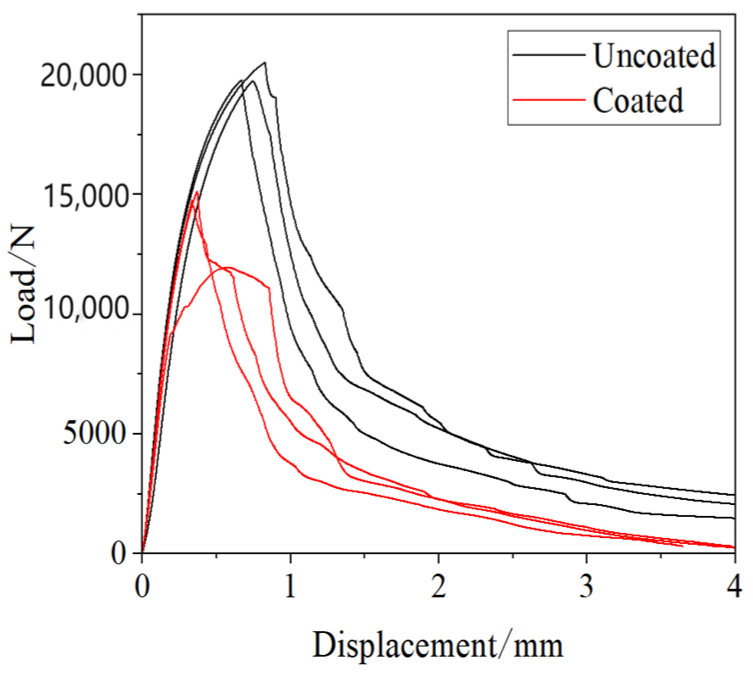
Quasi-static tensile test results.

Figure 15 shows the experimental results indicate that the strength of joints in uncoated samples is significantly higher than that of joints in galvanized samples. The average failure load for joints in uncoated samples is 20,003 N, while for galvanized samples it is only 13,937 N, representing a reduction of approximately 23.0%. Additionally, the failure displacement for galvanized samples occurs earlier compared to uncoated samples.

**Figure 16:**
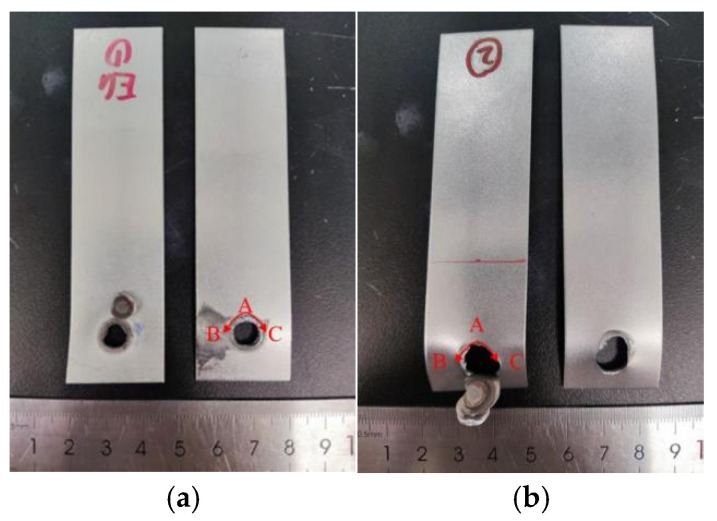
Fracture macroscopic morphology of TS specimen. (**a**) Galvanized specimens. (**b**) No galvanized layer.

Figure 16 shows the fracture failure images of both galvanized and uncoated samples, with the failure mode being button fracture in both cases. During the overall failure process, the fracture initiates at the tip of the plate interface, point A, and then propagates in the plate thickness direction and around the circumference on both sides of the molten pool at points B and C. In galvanized samples, the failure crack propagation leads to complete detachment of the weld core due to the embrittlement of the heat-affected zone caused by LME cracks. In contrast, uncoated samples exhibit some degree of plate tearing.

**Figure 17:**
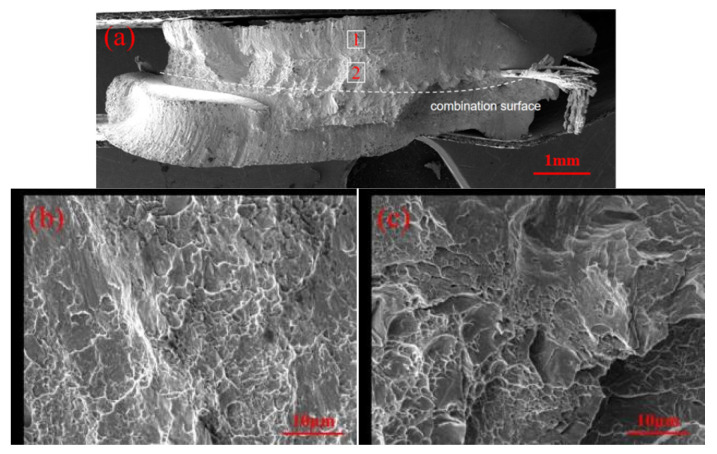
Fracture view of welded joints of non-zinc-plated specimens. (**a**) Overall shape of the fracture. (**b**) Enlarged view of area 1. (**c**) Enlarged view of area 2.

Figure 17 shows the fracture morphology of the weld points in uncoated samples. Figure 17a displays the overall fracture surface, which is irregular and includes two distinct fracture regions: one near the base material surface (Region 1) and another near the molten pool (Region 2). The fracture mode of the weld point is mixed. In Region 1, a significant number of river-like patterns can be observed, extending in the direction of plate fracture. In Region 2, irregular fan-shaped cleavage planes are visible near the interface, along with numerous fine dimples and white tear ridges. The failure crack initiates near the interface in this region and then propagates through the plate thickness and around the circumference, ultimately leading to brittle fracture in Region 1.

**Figure 18:**
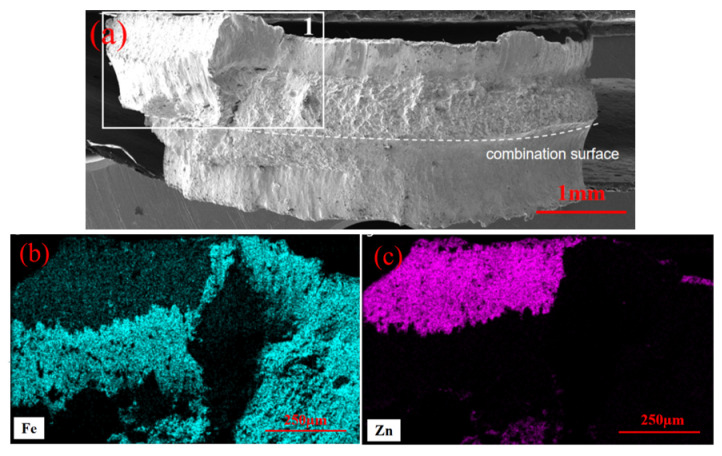
Micro-morphology and energy spectrum scanning of welded joints with galvanized layers. (**a**) Overall shape of the fracture. (**b**) Elemental scanning results of Fe in fracture zone 1. (**c**) Elemental scanning results of Zn in fracture zone 1.

Figure 18 shows the EDS elemental mapping results of the fracture surface for the galvanized samples, with a focus on a localized area of the fracture. The results indicate a significant accumulation of Zn elements at the fracture section, with the Zn distribution extending from the weld core surface into the interior of the plate. This finding confirms that LME cracks contributed to the fracture failure process in the TS samples.

**Figure 19:**
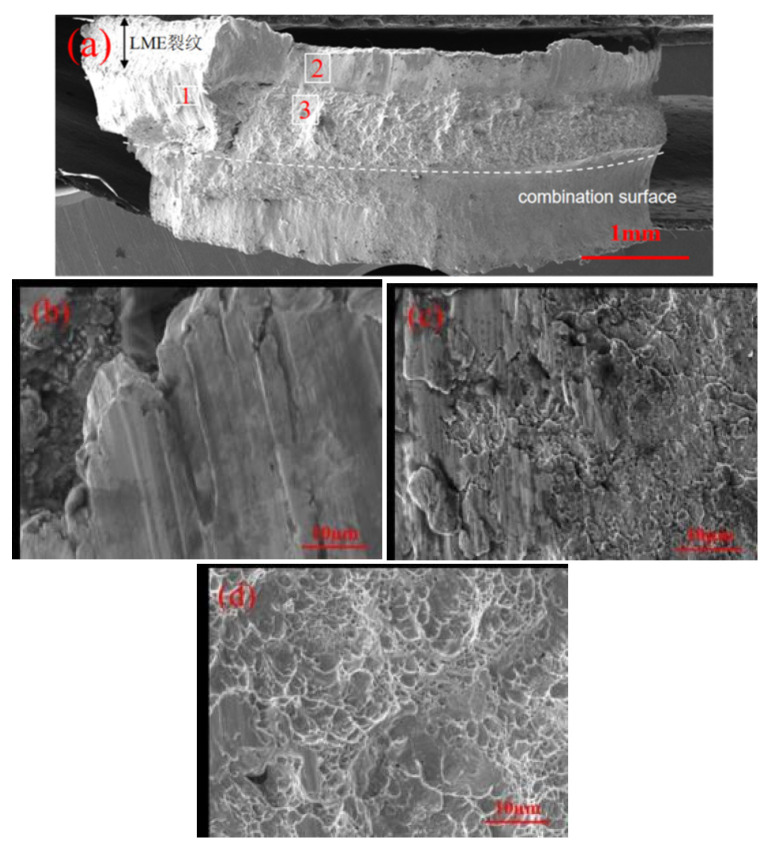
Morphology of welded joints of galvanized specimens. (**a**) Overall shape of the fracture. (**b**) Enlarged view of area 1. (**c**) Enlarged view of area 2. (**d**) Enlarged view of area 3.

Figure 19 shows the microscopic fracture morphology of the button fractures in galvanized samples. Based on the elemental mapping results and fracture patterns, the fracture region is divided into three parts: below the LME cracks (Region 1), near the plate surface (Region 2), and close to the interface (Region 3).

Overall, the fracture mode is primarily mixed. The failure crack initiates at the interface region. The magnified view of Region 1 shows a clear shear fracture morphology, indicating brittle fracture near the LME cracks. In Region 2, the magnified image displays river-like patterns with many small steps merging into larger steps. The direction of these river-like patterns aligns with the crack propagation direction, reducing the region’s ability to resist external loads. In Region 3, the magnified view reveals that the fracture mode is predominantly cleavage, featuring numerous fan-shaped cleavage planes along with some fine dimples. The involvement of LME cracks throughout the failure process reduces the load-bearing area around the weld core perimeter, accelerating the overall failure process and resulting in a lower failure load.

The strength of spot-welded joints is influenced by multiple factors. Since both types of samples use plates of the same thickness, the diameter of the molten pool significantly impacts joint strength. Given that the average molten pool diameter in galvanized samples is smaller than that in uncoated samples, it is necessary to exclude the effect of the molten pool diameter and assess whether the static strength of joints is consistent under the same molten pool diameter for both types of samples and whether LME cracks have any impact. Based on research on joint static strength in the literature [18,19], an approximate formula for calculating the strength of spot-welded joints under lap-shear loading conditions can be derived. This formula establishes the relationship between spot-welded joint failure modes and failure load, indicating that joint strength is closely related to material strength and the load-bearing area.

(2) $$F_{1F}=f_{1}D^{2}\tau$$

(3) $$F_{PF}=f_{2}Dt\tau_{t}$$

where

*F*_1*F*_ and *F_PF_* represent the failure loads for interface fracture and button fracture, in newtons (N);
f_1_ and f_2_ are material correction factors;
*D* is the diameter of the molten pool, measured in millimeters (mm);
t is the plate thickness, also measured in millimeters (mm);
σt denotes the tensile strength of the material, measured in megapascals (MPa);
*τ* is the shear strength of the material, typically taken as 0.577σt.

Substituting values into the formula reveals that for galvanized samples without LME cracks, the theoretical calculated value of 18,169 N is significantly higher than the experimental value of 13,937 N. The theoretical load value is reduced by approximately 9.1%, whereas the actual experimental value shows a reduction of 23.0%. This indicates that LME cracks have a significant impact on the static strength of the joints, which is consistent with the conclusions drawn from the experiments.

### 4.2. Static Tension Finite Element Modelling

Finite element modeling and simulation calculations of static tensile samples made of Q&P980 steel were performed using LS-DYNA software. Based on the dimensions of the static tensile test samples, corresponding plate models were established. At the center of the overlap region, metallographic measurements determined that the heat-affected zone widths for galvanized and uncoated samples were 1.18 mm and 0.95 mm, respectively. Using these measured widths, idealized models for the molten pool and heat-affected zone were created, with the heat-affected zone not subdivided. In this model, both the molten pool and the heat-affected zone are cylindrical in shape.

**Figure 20:**
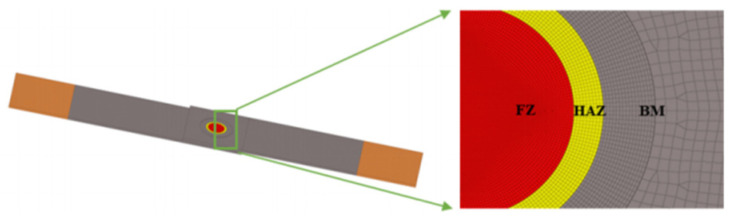
Finite element model of static tensile non-zinc-plated specimen and refinement of the central region.

To ensure accurate calculations for the molten pool and nearby areas, a refined mesh with a size of 0.1 mm was used within a 7 mm radius of the central region. For increased computational efficiency, the mesh size in the other plate areas was set to 0.3 mm. Figure 20 shows the finite element model of the uncoated static tensile sample and the refined mesh in the central region.

**Figure 21:**
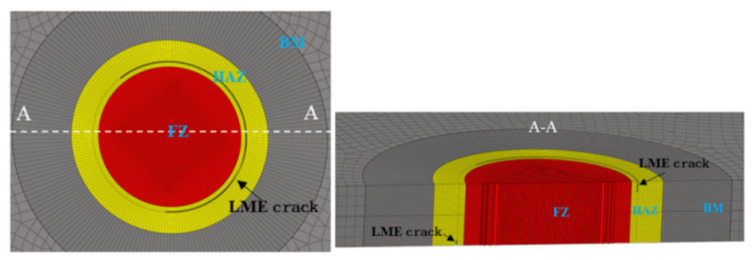
Centre view and section view of TS specimen with LME cracks.

On the basis of the original uncoated sample model, a static tensile model of galvanized samples containing LME cracks was created by removing mesh elements. To facilitate modeling while capturing the characteristics of the cracks, an approximate crack modeling method was proposed, using the average crack depth as the standard for LME crack modeling. Based on CT scan results, the positions and lengths of the cracks were determined and accurately incorporated into the finite element simulation model. The effect of the galvanized layer thickness was not considered in this model. For the crack width, referring to a relevant study [20] and mesh size, a value of 0.05 mm was chosen. This required further refinement of a single layer of mesh and deletion of the corresponding mesh elements to obtain the finite element static tensile model with LME cracks in the galvanized samples. Figure 21 shows the central view and a cross-sectional view of the finite element model containing LME cracks.

Due to temperature variations during the welding process, the microstructure of the material in different regions of the weld joint undergoes significant changes. These changes directly affect the material properties of each region, which in turn impacts the accuracy of the simulation model. Typically, material properties for each region are determined by cutting small samples from each region for performance evaluation. However, this method requires highly specialized testing equipment, is complex to operate, and incurs high costs, making it difficult to implement [21]. Cahoon et al. [22], through extensive theoretical research and practical engineering experience, established relationships between microhardness and tensile strength, as well as yield strength. These relationships are represented by the following Equations (4) and (5):

(4) $$\sigma =(1-\alpha )A{\left(\varepsilon_{p}+\varepsilon_{0}\right)}^{n}+\alpha (\sigma_{s}+C\varepsilon_{p}^{n})$$

(5) $$\sigma_{S}=\frac{H}{3}{\left(0.1\right)}^{n}$$

where

*H* represents the material microhardness, measured in HV (Vickers hardness);
Σb and *σ_s_* are the tensile strength and the yield strength, respectively, measured in MPa;
n is the material hardening index, which is considered a constant for materials.

**Figure 22:**
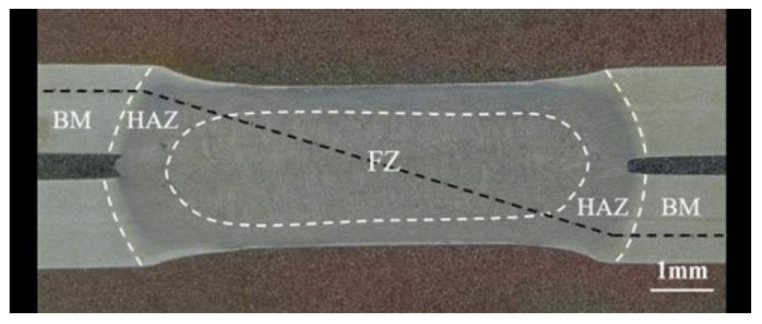
Q&P980 steel spot weld head hardness test route.

**Figure 23:**
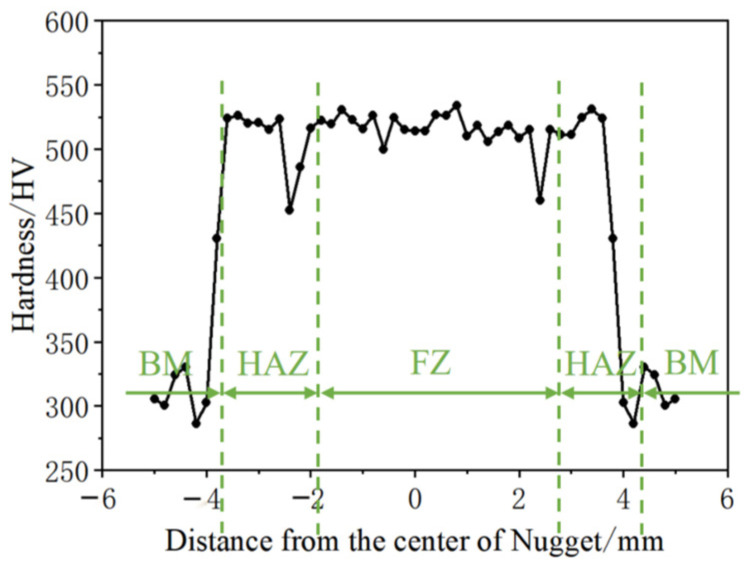
Q&P980 steel spot weld head hardness profile.

Figure 22 and Figure 23 show the microhardness testing paths and results for the molten pool (FZ), the heat-affected zone (HAZ), and the base material (BM) of the Q&P980 steel spot-welded joint under welding parameter 6 (welding current: 10 kA, welding time: 360 ms, electrode pressure: 3 kN). Hardness was tested at intervals of 0.02 mm. The results indicate that the hardness is highest in the molten pool region, with values remaining relatively constant from the center to the edges, averaging 518 HV. The average hardness in the heat-affected zone is 479 HV, with a noticeable decrease near the base material. In the base material region, hardness fluctuates less, with an average value of 309 HV.

**Figure 24:**
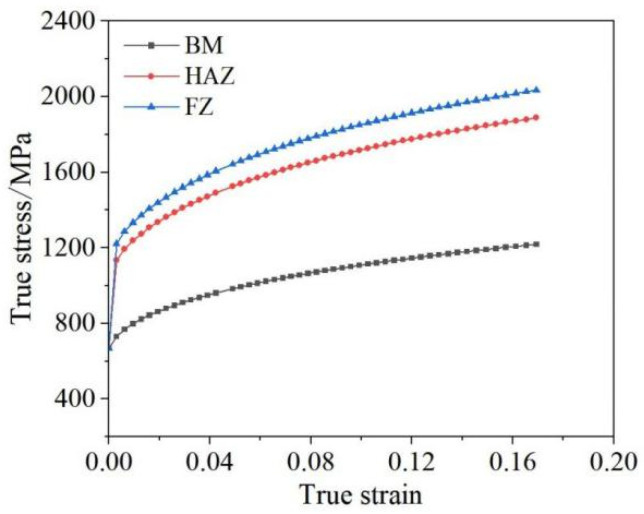
True stress–strain curves for each region of the Q&P980 steel spot weld head.

According to the hardness ratio of each region, the yield strength and the tensile strength of the molten core zone and the heat-affected zone can be approximated by combining the above formula, and the true stress–strain curves of the molten core zone and the heat-affected zone can be calculated according to the true stress–strain curves of the base metal in the test, as shown in Figure 24. The elastic modulus of each region is 205 GPa, the density is 7750 Kg/m^3^, and the Poisson ratio is 0.31.

Since the molten pool region is relatively hard and no interface failure was observed in the tests, failure in the molten pool region is not considered. For base material failure, the relationship between stress triaxiality and fracture strain from the previously mentioned Gissmo material failure model is used. Given the complexity and high cost of material testing in the heat-affected zone, the failure relationship curve for the heat-affected zone is determined by scaling the base material failure curve. The scaling factors were 0.11 for galvanized samples and 0.21 for uncoated samples.

**Figure 25:**
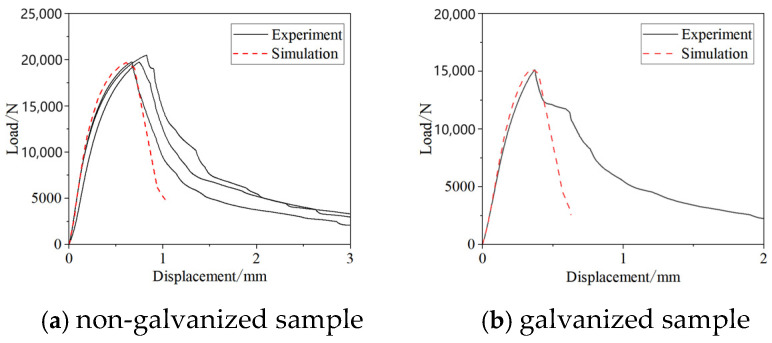
Comparison of TS specimen load–displacement results.

The established TS sample models include both uncoated samples and galvanized samples with LME cracks. For the galvanized samples, one specific sample containing LME cracks was selected for study. Figure 25a shows a comparison between the finite element simulation model and the experimental load–displacement curves. The finite element simulation results closely match the experimental data, demonstrating a similar failure trend. The failure load obtained from the finite element analysis is 19,700 N, which is very close to the experimental average failure load of 20,003 N, with only a 1.5% difference. It shows that the finite element model of the non-galvanized sample simulated the fracture process of the spot-welded joint well. Figure 25b presents a comparison between the finite element results and the experimental data for the galvanized sample with LME cracks. The curves show good consistency, with the maximum tensile failure load from the finite element analysis being 15,200 N, which differs by less than 1% from the experimental results. This confirms that the finite element model is accurate and reliable, accurately reflecting the fracture process. In the tensile test, the object breaks after reaching the tensile strength, but the fracture is not instantaneous; it occurs gradually as the force decreases until it is completely torn. This is a feature of the experimental process. In the simulation, however, we focused on the failure process and its subsequent states. Because the simulation was a simplified process, it was difficult to accurately simulate the gradual fracture observed in the experiment. Therefore, there are some differences between the simulated image and the experimental image after reaching the peak.

**Figure 26:**
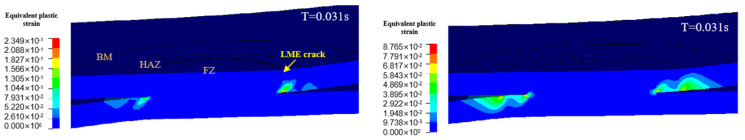
Equivalent plastic strain maps in the pre-crack initiation stage.

**Figure 27:**
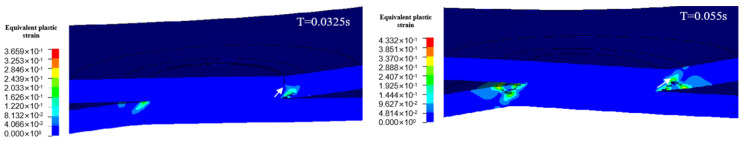
Cloud view of equivalent plastic strain during crack initiation phase.

**Figure 28:**
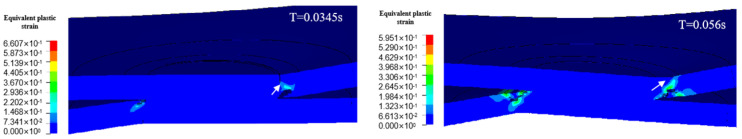
Equivalent plastic strain maps in the middle stage of crack initiation.

**Figure 29:**
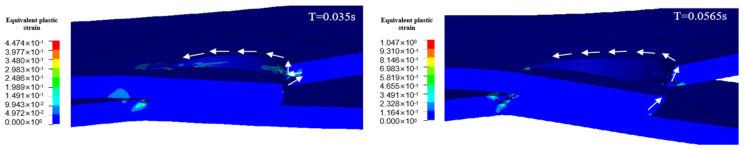
Equivalent plastic strain cloud at the late stage of crack initiation.

The tensile process of spot-welded joints is a dynamic process, and finite element simulation results are used to replace the deformation and fracture process of spot-welded samples in experiments. To better simulate and compare the fracture processes, the evolution of equivalent plastic strain during fracture was analyzed for both galvanized samples and uncoated samples with the same weld nugget diameter. Figure 26, Figure 27, Figure 28 and Figure 29 show the comparison of equivalent plastic strain during the fracture process of the joints for both galvanized samples with LME cracks (left) and uncoated samples (right). To clearly illustrate the fracture process, four stages were selected: pre-crack initiation, crack initiation, mid-crack propagation, and post-crack propagation.

The comparison diagrams reveal that while the overall fracture trends of the two types of samples are similar, there are notable differences in the crack propagation paths. Under the lap shear loading, the stress concentration at the edge of the weld nugget first reaches the yield stage and is also the area of concentrated plastic strain, where the failure crack initiates. The crack then propagates both along the plate thickness and around the weld nugget circumference. During crack propagation, the presence of LME cracks in the heat-affected zone significantly impacts the local plastic strain region, causing the failure crack path to extend towards the LME cracks. In contrast, the uncoated sample maintains its original propagation path in the plate thickness direction. The presence of LME cracks accelerates the fracture process, leading to earlier crack initiation and failure in the galvanized sample compared to the uncoated sample. The uncoated sample, lacking pre-existing LME cracks, can endure more deformation, resulting in significantly greater deformation and higher load-bearing capacity, thereby absorbing more fracture energy.

**Figure 30:**
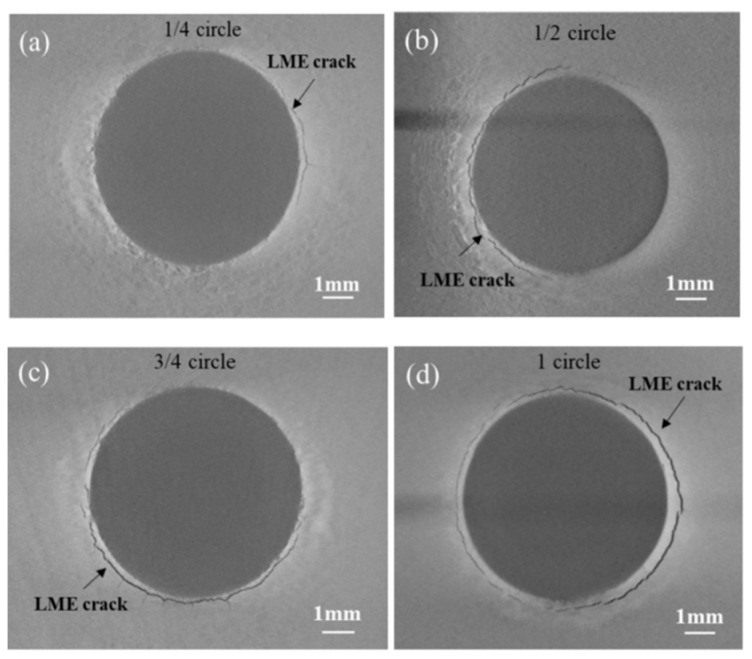
Approximation of crack length on LME cracked surfaces.(**a**) One-quarter. (**b**) Two-quarters. (**c**) Three-quarters. (**d**) The full circumference.

To investigate the impact of LME cracks with varying positions and sizes on the strength of spot-welded joints, a detailed study was conducted. The CT scan results reveal that LME cracks typically occur within a radius of approximately 3.5 mm. Due to uncertainties in the depth of LME cracks, cracks with average depths of 0.5 mm, 0.8 mm, and 1 mm were considered for this study. Additionally, the length of surface cracks in LME also needed to be evaluated. Figure 30 illustrates the approximate lengths of surface cracks as detected by CT scans, which are categorized as one-quarter, two-quarters, three-quarters, or the full circumference of the weld. The probabilities of these lengths occurring are 30.4%, 21.7%, 30.4%, and 17.4%, respectively. Utilizing the previously validated LME crack model for galvanized samples, finite element simulations were employed to assess how varying severities of LME cracks affect the tensile shear strength of the weld joints.

**Figure 31:**
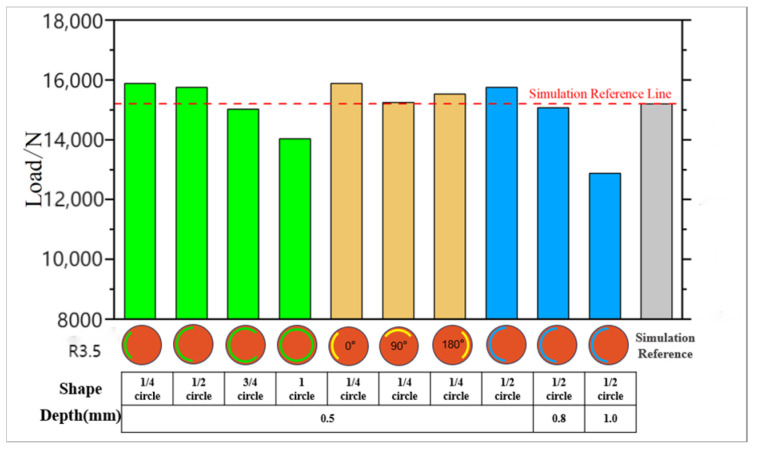
Joint strength statistics for different LME crack locations and sizes.

Figure 31 shows the finite element results of spot-welded joints with cracks of varying sizes and positions. The simulations were conducted using the static tensile model with LME cracks as the reference, with modifications made to the LME crack sizes and positions. For cracks of the same diameter, as the crack circumference length increases from one-quarter of the circumference to a full circumference, the failure load of the joint decreases from 15,866 N to 14,031 N. This indicates that the longer the crack circumference length, the more pronounced the reduction in joint strength. For pre-existing cracks with a one-quarter circumference, the strength variations of the joint were studied under three different crack orientations: 0°, 90°, and 180°. The impact of the crack at the 90° orientation was found to be greater than in the other two orientations. For cracks at the same circumferential position, the depth of the crack also significantly affected joint strength. The results show that as the crack depth increases, the joint strength decreases significantly, from 15,756 N at a 0.5 mm crack depth to 12,873 N at a 1.0 mm crack depth.

### 4.3. Fatigue Behaviour Analysis of Spot-Welded Joints

**Figure 32:**
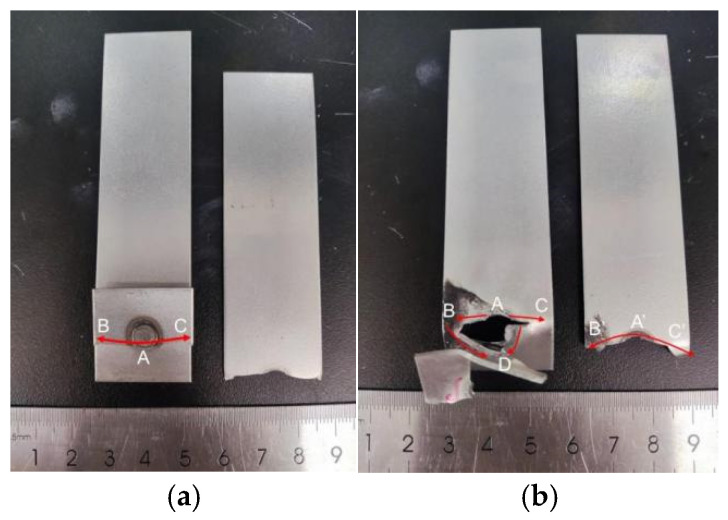
Failure diagram for fatigue test with and without galvanized specimens. (**a**) No galvanized specimens. (**b**) Galvanized specimens.

Based on the static tensile test results, fatigue tests were conducted using a load amplitude of 1800 N. Table 6 presents the fatigue test results for TS (non-galvanized) and CP (galvanized) specimens. The average fatigue life of the galvanized spot-welded joints was 20,892 cycles, which is significantly lower than the average fatigue life of 41,403 cycles for non-galvanized joints, representing a reduction in fatigue life of nearly 50%. Both galvanized and non-galvanized specimens exhibited eyebrow-shaped fractures as the failure mode. However, the galvanized specimens displayed multiple instances of sheet metal tearing, which may be related to the presence of liquid metal embrittlement (LME) cracks. Figure 32 illustrates the fracture failure patterns for both types of sheet metal.

For the eyebrow-shaped fracture failure in galvanized specimens, fatigue crack initiation occurs at point A, the high-stress notch tip, typically associated with microscopic defects or inclusions. The crack propagates through the sheet thickness and extends in the direction perpendicular to the tensile stress, simultaneously expanding at points B and C. When the crack encounters the liquid metal embrittlement (LME) crack region during propagation, its path deviates. One portion continues to expand along the sheet width, while another propagates along the circumference of the LME crack fusion zone (point D). Under sustained cyclic loading, crack growth becomes increasingly pronounced along both paths, ultimately leading to failure either through fatigue crack propagation to the sheet edge or by button pull-out. The presence of LME cracks alters the crack propagation direction, progressively reducing the load-bearing area of the joint and accelerating fatigue crack growth. In contrast, for non-galvanized specimens exhibiting eyebrow-shaped fractures, fatigue crack initiation also occurs near the high-stress notch tip (point A). However, the fatigue crack propagates directly through the sheet thickness and expands perpendicularly to the tensile stress direction simultaneously at points B and C. Due to the absence of LME cracks, the fatigue crack maintains its original direction under continued cyclic loading until it reaches the sheet edge, completing the failure process.

**Figure 33:**
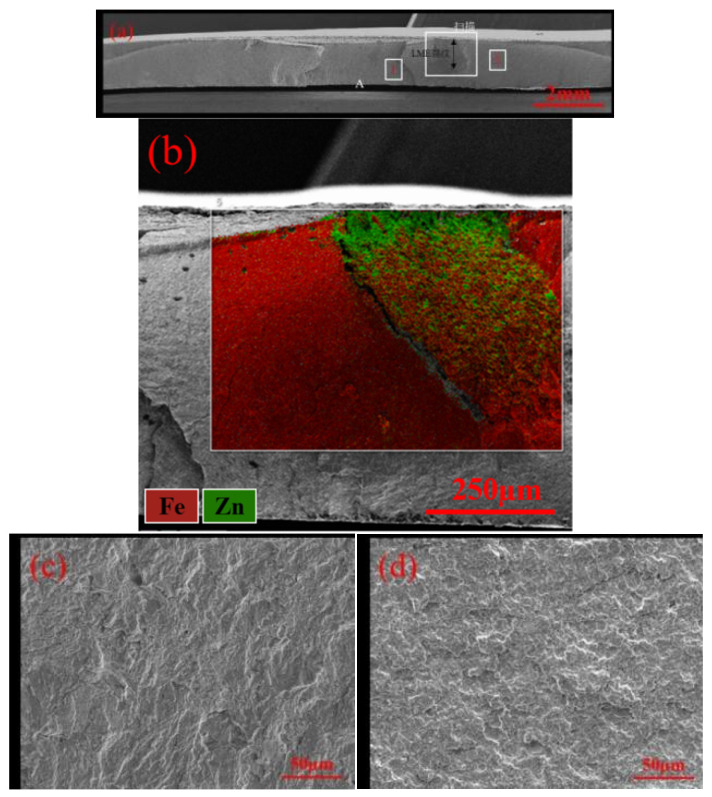
Fatigue test fracture of galvanized specimens. (**a**) Overall shape of the fracture. (**b**) Regional Energetic Elemental Scanning. (**c**) Local enlargement of position 1. (**d**) Area 2 local zoom.

The fatigue fracture surfaces of both specimen types exhibit similar morphologies. Figure 33 illustrates the fatigue test fracture surface of a galvanized specimen. Figure 33a shows the overall fracture surface morphology, with fatigue crack initiation at the high-stress region A, followed by propagation through the sheet thickness and width. Energy-dispersive spectroscopy (EDS) elemental scanning was performed in the scanned area. Figure 33b reveals that the fatigue crack propagation path intersects with LME cracks. Figure 33c,d provide magnified views of the fusion zone circumference (area 1) and the sheet metal region (area 2), respectively. Compared to the sheet width direction, the circumferential direction exhibits more pronounced tear ridges in fatigue crack propagation, indicating brittle fracture associated with the brittle martensite near the joint. In area 2, farther from the joint, the martensite is finer, resulting in less pronounced tear ridges.

**Figure 34:**
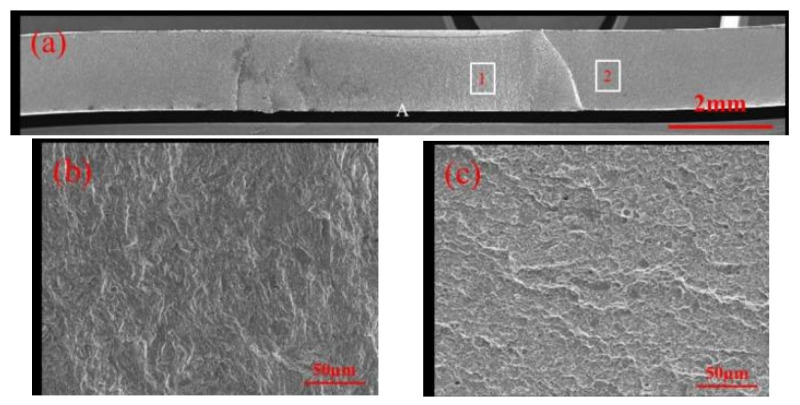
Fatigue test fracture without galvanized specimen. (**a**) Overall shape of the fracture. (**b**) Local enlargement of position 1. (**c**) Area 2 local zoom.

Figure 34 depicts the fatigue test fracture surface of a non-galvanized specimen. This fracture surface closely resembles that of the galvanized specimen, with the primary difference being the absence of LME crack regions. Figure 34a shows the overall fracture surface morphology, with fatigue crack initiation also occurring at the high-stress region A, followed by propagation through the sheet thickness and width. Figure 34b,c provide magnified views of the fusion zone circumference (area 1) and the sheet metal region (area 2), respectively. Similarly, more pronounced fatigue fracture tear ridges can be observed in the circumferential direction, with the entire fracture process also characterized by brittle failure.

## 5. Conclusions

(1) Spot welding experiments on galvanized Q&P980 steel revealed that liquid metal embrittlement (LME) cracks occurred exclusively in galvanized specimens, while non-galvanized specimens showed no evidence of LME cracking. This observation indicates that the presence of a zinc coating is a crucial factor in the formation of LME cracks. These cracks are predominantly located above the fusion zone and at the heat-affected zone (HAZ) step. Energy-dispersive spectroscopy (EDS) analysis demonstrates that the zinc content decreases with increasing crack depth.

(2) Quasi-static tensile tests and fatigue tests conducted on tensile–shear (TS) specimens indicated that LME cracks significantly impact the mechanical properties of spot-welded joints. Examination of failure fracture surfaces revealed striking similarities between galvanized and non-galvanized specimens, despite the presence of LME cracks in the former.

(3) Finite element simulations of quasi-static tensile loading demonstrated that LME cracks influence the stress and strain field evolution during the fracture process. These cracks promote failure crack propagation in the LME crack direction, thereby accelerating the overall failure process. The impact of LME cracks on joint static strength varies with crack severity. As the circumferential length and depth of the cracks increase, the static strength of the joint decreases. Furthermore, the influence of LME cracks differs based on their location, with cracks at the 90° orientation exhibiting the most significant impact.

## Figures and Tables

**Table 1:** Q&P980 chemical composition of steel (mass fraction, %).

Element	C	Si	Mn	S	Ti	Cr	Cu	Ni	Fe
Mass Fraction (%)	0.2	2.0	2.29	0.001	0.01	0.26	0.01	0.02	0.95

**Table 2:** Q&P980 mechanical properties of steel.

Elastic Modulus/GPa	Tensile Strength/MPa	Yield Strength/MPa	Elongation/%
209	1023	669	24

**Table 3:** Welding parameter in resistance spot welding tests.

Welding Current/kA	Squeeze Time/ms	Weld Time/ms	Hold Time/ms	Electrode Force/kN
10	500	360	250	3

**Table 4:** Statistical table of LME crack location radius for galvanized samples.

Sample Number	Top Plate Crack Location Diameter/mm	Bottom Plate Crack Location Diameter/mm
1	6.9	6.8
2	7.6	7.5
3	6.8	6.8

**Table 5:** Failure strain at different stress triaxiality values.

Specimen Type	Stress Triaxiality	Failure Strain
Uniaxial Tensile Specimen	0.15	0.7904
Shear Specimen	0.33	0.7184
R5 Notched Specimen	0.49	0.7535
R3 Notched Specimen	0.54	0.9742

**Table 6:** Fatigue test results.

Test No.	Galvanized Sample		Non-Galvanized Sample	
	Fatigue Life (cyc)	Average Life (cyc)	Fatigue Life (cyc)	Average Life (cyc)
1	21,053		50,637	
2	20,438	20,892	40,997	41,403
3	21,185		32,575	

## Data Availability

The original contributions presented in the study are included in the article, further inquiries can be directed to the corresponding author.

## References

[1] Wang X. (2021). An investigation of the progress of automotive lightweighting in China in the past five years. Automot. Dig..

[2] Tang J., Zou D., Jiang H., Chen X., Li S. (2014). Experimental study on fatigue performance of resistance spot welding process and lap joints of Q&P steel. Therm. Process. Technol..

[3] Mukai Y. (2005). The development of new high-strength steel sheets for automobiles. Kobelco Technol. Rev..

[4] Hernandez B.V.H., Kuntz M.L., Khan M.I., Zhou Y. (2008). Influence of microstructure and weld size on the mechanical behaviour of dissimilar AHSS resistance spot welds. Sci. Technol. Weld. Join..

[5] Ina K., Koizumi H. (2004). Penetration of liquid metals into solid metals and liquid metal embrittlement. Mater. Sci. Eng. A.

[6] Górka J., Suder W., Kciuk M., Stano S. (2023). Assessment of the Laser Beam Welding of Galvanized Car Body Steel with an Additional Organic Protective Layer. Materials.

[7] Marashi P., Pouranvari M., Sanaee S.M.H., Abedi A., Abootalebi S.H., Goodarzi M. (2008). Relationship between failure behaviour and weld fusion zone attributes of austenitic stainless steel resistance spot welds. Mater. Sci. Technol..

[8] Digiovanni C., Biro E., Zhou N.Y. (2019). Impact of liquid metal embrittlement cracks on resistance spot weld static strength. Sci. Technol. Weld. Join..

[9] Ge X. (2021). Study on the Effect of Liquid Metal Embrittlement Cracking on the Mechanical Properties of Resistance Spot Welded Joints of Zinc Bonded Q&P1180 Steel. Master’s Thesis.

[10] Choi D., Uhm S., Enloe C., Lee H., Kim G., Horvath C. (2017). Liquid metal embrittlement of resistance spot welded 1180trip steel-effects of crack geometry on weld mechanical performance. Mater. Sci. Technol..

[11] DiGiovanni C., Han X., Powell A., Biro E., Zhou N.Y. (2019). Experimental and numerical analysis of liquid metal embrittlement crack location. J. Mater. Eng. Perform..

[12] Kim Y.G., Kim I.J., Kim J.S., Chung Y.I., Choi D.Y. (2014). Evaluation of surface crack in resistance spot welds of zn-coated steel. Mater. Trans..

[13] Jang G.H., Kwon K., Kim W., Uhm S., Lee T., Lee C.S. (2021). Effect of type-b liquid metal embrittlement cracks on high-cycle fatigue properties of spot-welded 1180 TRIP steel. Sci. Technol. Weld. Join..

[14] Wang X., Xie Y., Liu Z., Sun Q., Shen X., Zhang Q., Hu Z., Misra R.D.K. (2022). Zn-induced liquid metal embrittlement and mechanical properties of advanced high-strength steel with resistance spot weld. Mater. Sci. Eng. A.

[15] Kwon K., Jang G., Kim W., Uhm S., Lee T., Lee C.S. (2021). Effect of Type-C liquid metal embrittlement on mechanical properties of spot-welded TRIP steel. J. Mater. Res. Technol..

[16] Dong Y., Xue R., Zhang Q., Chen Y., Wu H. (2023). Characterization of dynamic mechanics and fracture-failure behavior of high-strength duplex steels. Hebei Metall..

[17] Mohr D., Marcadet S.J. (2015). Micromechanically-motivated phenomenological Hosford–Coulomb model for predicting ductile fracture initiation at low stress triaxialities. Int. J. Solids Struct..

[18] Sun X., Stephens E.V., Davies R.W., Khaleel M.A., Spinella D.J. (2004). Effects of Fusion Zone Size on Failure Modes and Static Strength of Aluminum Resistance Spot Welds. Weld. J..

[19] Shi Y. (2013). Research on Fatigue Performance and Life Prediction Method of Aluminium Alloy Spot Welded Joints. Ph.D. Thesis.

[20] Ma Y., Yu Y., Geng P., Ihara R., Maeda K., Suzuki R., Suga T., Ma N. (2021). Fracture modeling of resistance spot welded ultra-high-strength steel considering the effect of liquid metal embrittlement crack. Mater. Des..

[21] Li X. (2023). Study on Crack Extension and Fatigue Life of Spot Welded Joints of High Strength Steel Under Residual Stress. Master’s Thesis.

[22] Cahoon J. (1972). An improved equation relating hardness to ultimate strength. Metall. Mater. Trans. B.

